# Trend and treatment outcomes of latent tuberculosis infection among migrant persons in Japan: retrospective analysis of Japan tuberculosis surveillance data

**DOI:** 10.1186/s12879-020-05712-1

**Published:** 2021-01-09

**Authors:** Lisa Kawatsu, Kazuhiro Uchimura, Akihiro Ohkado

**Affiliations:** Department of Epidemiology and Clinical Research, The Research Institute of Tuberculosis, 3-1-24, Matsuyama Kiyose, Tokyo, Japan

**Keywords:** Latent tuberculosis infection, Migrants, Trend, Treatment outcome

## Abstract

**Background:**

Screening for latent tuberculosis infection (LTBI) among migrant population has become a critical issue for many low tuberculosis (TB) burden countries. Evidence regarding effectiveness of LTBI programs are limited, however, partly because of paucity of national data on treatment outcomes for LTBI. In Japan, notification of LTBI is mandatory, and its treatment outcome is reported as part of Japan’s national TB surveillance system. We thus conducted a detailed analysis of LTBI among foreign-born persons, to update the epidemiological trend of newly notified LTBI between 2007 and 2018, and to examine the treatment regimen and outcome of those notified in 2016 and 2017, focusing specifically on the potential risk factors for lost to follow-up.

**Methods:**

We extracted and analyzed the data of newly notified LTBI patients from the Japan Tuberculosis Surveillance System to examine the overall trend of notification and by age groups and modes of detection between 2007 and 2018, and the cohort data for treatment regimen and outcomes of foreign-born persons notified with LTBI in 2016 and 2017. Trends and proportions were summarized descriptively, and logistic regression analysis was conducted to identify potential risk factors for lost to follow-up. Comparisons were made with the Japan-born patients where appropriate, using chi-squared tests.

**Results:**

Both the number and proportion of LTBI among foreign-born persons have been constantly increasing, reaching 963 cases in 2018. Cohort analysis of the surveillance data indicated that the proportion of those on shorter regimen was higher among the foreign- than Japan-born patients (5.5% vs. 1.8%, *p* < 0.001). The proportion of those who have been lost to follow-up and transferred outside of Japan combined was higher among the foreign- than Japan-born patients (12.0% vs, 8.2%, *p* < 0.001). Risk factors for lost to follow-up were being employed on a temporal basis, and job status unknown (adjusted odds ratios 3.11 and 4.09, 95% confidence intervals 1.34–7.26 and 1.60–10.48, respectively).

**Conclusions:**

Migrant population face greater risk of interrupting LTBI treatment, and interventions to improve adherence are a critical component of programmatic management of LTBI. Further studies are needed to explore the cultural and socioeconomic situation in which foreign-born persons undergo LTBI treatment in Japan.

## Background

Foreign-born populations contribute considerably to both the number and proportion of all tuberculosis (TB) cases, especially in countries with low TB incidence [1–3]. In Japan as well, which is a TB middle-burden country with a notification rate of 12.3 per 100,000 in 2018, although the proportion of foreign-born persons out of all TB cases was 9.5%, that among those aged between 15 and 24 years old has reached 70.8% in the same year [4]. It has been pointed out that the majority of these TB cases occurs due to the reactivation of latent tuberculosis infection (LTBI) acquired in their country of origin. As a large proportion of migrants is born in high-TB burden countries, where it has been estimated that approximately 26 to 46% of the population are latently infected with TB [5, 6], screening for not only active TB but also LTBI among the migrants, has become a critical issue for many low TB burden countries (< 10 cases/100,000 population). In Japan as well, 58.2% of foreign-born TB patients come from 5 countries in Southeast Asia, with a TB incidence of more than 100 per 100,000 population [4].

Yet in its recently revised guideline, the World Health Organization (WHO) has only conditionally recommended LTBI screening among migrants living in low TB burden countries [7]. The guideline points to the low-quality evidence for the effectiveness and cost-effectiveness of LTBI programs in these settings, including challenges in achieving treatment adherence and completion rate. Studies reporting national data on treatment outcomes for LTBI are however limited, due mainly to LTBI not requiring mandatory notification in many countries.

In Japan, however, when a physician diagnoses LTBI and determines that treatment is necessary, he or she is required to notify the local public health center, just as is the case with active TB, under the Infectious Diseases Control Law. Once notified, treatment outcome is evaluated and reported as part of Japan’s national TB surveillance system (JTBS) [4]. There is no national guideline that indicates clear priorities for targeted LTBI screening, however, approximately 60% of notified LTBI patients are detected via contact investigation [4]. Treatment is strongly recommended for selected populations considered to be at a higher risk of developing TB, including people living with HIV/AIDS, silicosis, people on treatment with immunosuppressants, and those who have been recently infected [8].

The authors have previously estimated the treatment outcomes of LTBI notified between 2007 and 2014 – the outcomes were however determined using proxy variables because the JTBS then only evaluated treatment outcome for pulmonary TB [9]. In 2017, the JTBS underwent a system revision, making cohort analysis possible for all types of TB, including extra-pulmonary TB and LTBI, for notifications from 2017 and onward. We thus conducted a detailed analysis of LTBI among foreign-born persons, with two main objectives; firstly to provide a detailed description of the epidemiological trend of newly notified LTBI between 2007 and 2018, and secondly, to examine the treatment regimen and outcome of those notified in 2016 and 2017, focusing specifically on the potential risk factors for lost to follow-up among foreign-born LTBI patients, with the ultimate goal of providing epidemiological evidence which can be used to guide decision-making on screening and testing policies for LTBI among foreign-born persons in Japan.

## Methods

Japan introduced its first electronic surveillance system in 1987, which since then has undergone several major system revisions. Details of the system can be found elsewhere [4]. Briefly, at the end of each year, it produces four sets of data; 1) a list of all active TB and LTBI cases newly notified in that year (“newly notified dataset”), 2) a list of all active TB and LTBI cases registered at the end of the year (“end of year dataset”), 3) a list of active TB and LTBI cases notified in the previous year, with treatment outcomes (“cohort dataset”), and 4) a list of all cases who were de-registered in that year (“de-registered dataset”).

### Study population and data source

In order to examine the overall trend of notification and by age groups and modes of detection, we extracted the data of LTBI patients newly notified between 2007 and 2018, from the “newly notified dataset”. In order to analyze the treatment regimen and treatment outcomes, we extracted the cohort data of foreign-born LTBI patient who had been notified between 2016 and 2017 from the “cohort dataset”.

### Data analysis

Trends and proportions were summarized descriptively, and comparisons were made with the Japan-born patients where appropriate, using chi-squared analysis. Treatment outcomes were analyzed for those who have started treatment, as according to the outcome variables of the JTBS i.e. “treatment success”, “died”, “treatment failed”, “lost to follow-up”, “transferred-out”, “still on treatment” and “unknown”. From the variables that are collected via the surveillance, potential reasons for lost to follow-up were further explored, and logistic regression analysis was conducted to identify risk factors. The crude odds ratio (OR) and adjusted odds ratio (AOR) were reported, with their corresponding 95% confidence interval and *p*-values. The definitions for the main variables can be found in the Annex (S1 Appendix). R version 3.6.3 (R Development Core Team, Vienna, Austria) was used for all statistical analyses.

## Results

### Trend in LTBI notification

**Fig. 1:**
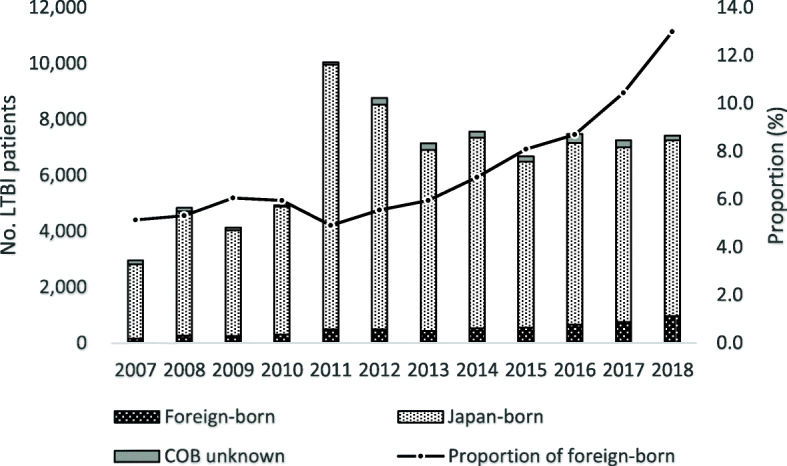
Annual trend of LTBI notification by country of birth, and proportion of foreign-born patients, 2007–2018

**Fig. 2:**
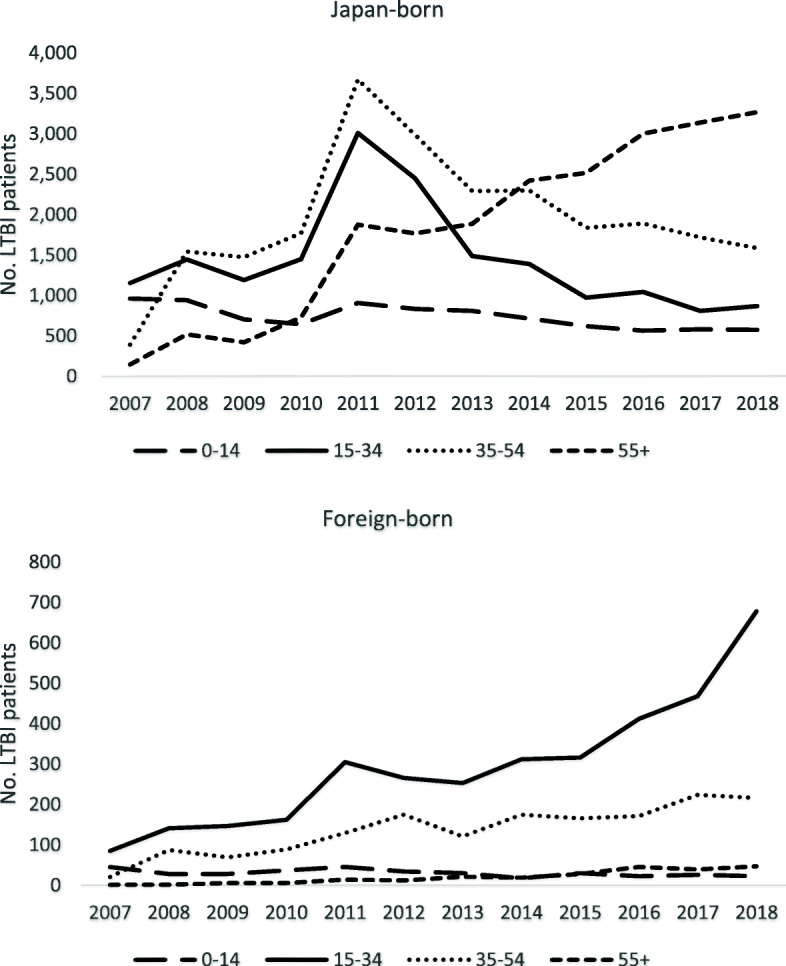
Trend of LTBI notification by country of birth and age groups, 2007–2018

**Fig. 3:**
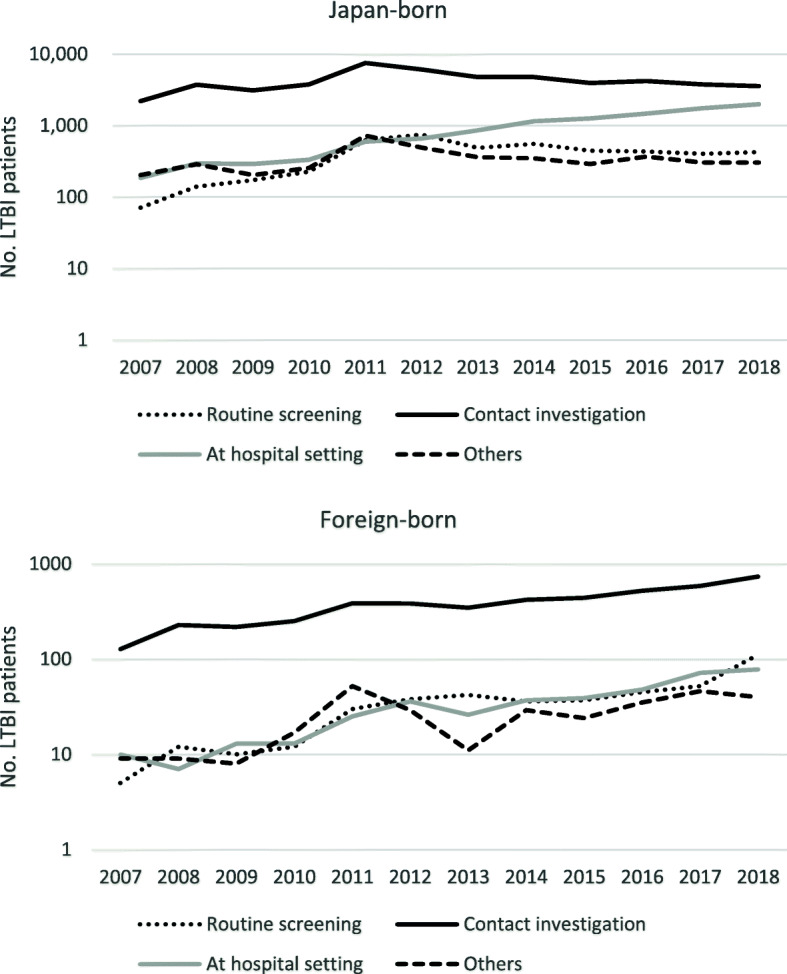
Trend of LTBI notification by mode of detection, 2007–2018

Figure 1 shows the annual trend of the number of overall LTBI notifications by country of birth, and the proportion of foreign-born persons among the total cases. The number of total LTBI notifications has reached a peak in 2011, and since then it has declined and in the past five years or so, has stabilized around 7000 a year. However, both the number and the proportion of foreign-born LTBI patients have increased steadily, reaching 963 cases in 2018 (13.0%, 963/7414). Looking at the trend in the notification by age groups, while among the Japan-born patients, the number of notifications has increased only among those aged 55 years old and above, among the foreign-born patients, the number has increased rapidly among all age groups except those aged between 0 and 14 years old (Fig. 2). Regarding the modes of detection, for both Japan and foreign-born patients, the majority were detected via contact investigation over the study period (foreign-born; 80.3%, and Japan-born; 72.3%). However, while among the Japan-born, only the number of those detected at hospital has increased, among the foreign-born, the numbers of those detected via contact investigation, routine screening, and at hospital all similarly expressed steady increase (Fig. 3).

### LTBI treatment regimen

From the “cohort dataset”, treatment regimen and outcome were available for 12,817 Japan-born and 1418 foreign-born LTBI patients. Of the 12,817 Japan-born and 1418 foreign-born LTBI patients, 96.0% (*n* = 12,298) and 92.3% (*n* = 1309) had started their treatment with INH monotherapy, respectively. Higher proportion of foreign-born than Japan-born patients had started their treatment with RFP monotherapy (5.5% vs. 1.8%, *p* < 0.001) (Table 1).

**Table 1:** Treatment regimen of LTBI patients upon registration (2016–2017)

	Japan-born		Foreign-born	
	n	%	n	%
**INH monotherapy**	12,298	96.0	1309	92.3
**RFP monotherapy**	225	1.8	78	5.5
**No treatment**	235	1.8	27	1.9
**Others**^**a**^	59	0.5	4	0.3
**TOTAL**	12,817	100.0	1418	100.0

*INH* isoniazid, *RFP* rifampicin ^a^“Others” consist of all other treatment categories, excluding “no treatment” – please see Annex (S1 Appendix)

### LTBI treatment outcome

Treatment outcomes of those who have started treatment, i.e. excluding those whose treatment regimen was recorded as “no treatment”, are summarized in Table 2. The overall treatment success was slightly higher among the Japan-born than foreign-born patients (85.8% vs. 82.2%, *p* < 0.001). While the proportion of lost to follow-up (8.2% vs. 8.4%, *p* = 0.78) were similar among the Japan-and foreign-born patients, the proportion of transferred-out was much higher among the foreign- than Japan-born patients (8.3% vs. 1.5%, p < 0.001). However, the “transfer-out” refers to a move outside the jurisdiction of the public health center to where the patient was initially notified, including both a move to another jurisdiction within Japan, and a move to another country. A closer analysis has revealed that of patients who have transferred-out, while among the Japan-born, 94.8% (183/193) were transfer-out within Japan, among the foreign-born, 43.0% (50/116) had transferred out of Japan. Considering that treatment outcome of those who have transferred outside of Japan is practically non-traceable, these were re-categorized as lost to follow-up – this had the effect of making lost to follow-up considerably higher among the foreign- than Japan-born patients (12.0% vs, 8.2%, *p* < 0.001).

**Table 2:** Treatment outcomes of LTBI patients (2016–2017), by county of birth

	Japan-born		Foreign-born	
	n	%	n	%
**Treatment success**	10,798	85.8	1142	82.2
**Died**	310	2.5	1	0.1
**Failed**	41	0.3	5	0.4
**Lost to follow-up**	1026	8.2	117	8.4
**Transferred-out**	193	1.5	116	8.3
within Japan	183	–	63	–
outside Japan	8	–	50	–
unknown	2	–	3	–
**Still on treatment**	132	1.0	8	0.6
**Unknown**	82	0.7	2	0.1
**TOTAL**	12,582	100.0	1391	100.0
Lost to follow-up, recategorized^a^	1034	8.2	167	12.0

^a^Sum of Lost to follow-up and Transferred outside of Japan

### Risk of lost to follow-up within foreign-born patients

Proportions of lost to follow-up and those who have transferred out of Japan (*n* = 167), among various socio-demographic characteristics within foreign-born persons are summarized in Table 3.

**Table 3:** Proportions of “lost to follow-up” among various characteristics, foreign-born LTBI patients (*n* = 1391)

	Total	LTFU	Transferred out of Japan	LTFU + transferred out of Japan	% of LTFU + transferred out of Japan
**Sex**					
Male	665	58	19	77	11.6
Female	726	59	31	90	12.4
**Age groups (years)**					
0–14	49	3	2	5	10.2
15–19	61	4	1	5	8.2
20–29	630	47	21	68	10.8
30–39	302	29	17	46	15.2
40–49	195	21	6	27	13.8
50 plus	154	13	3	16	10.4
**Job status**					
Healthcare workers (HCW)	110	10	0	10	9.1
Full-time work, other than HCW	495	28	24	52	10.5
Self-employed	19	1	1	2	10.5
Employed on daily/temporal basis	114	19	5	24	21.1
Houseworkers	16	2	0	2	12.5
Students, high school and above	432	38	9	47	10.9
Secondary, primary school students, other children and infants	54	4	2	6	11.1
Others and unknown	67	8	8	16	23.9
Unemployed	84	7	1	8	9.5
**Country of Birth**					
the Philippines	320	33	11	44	13.8
China	268	14	18	32	11.9
Vietnam	196	19	8	27	13.8
Nepal	194	17	0	17	8.8
Indonesia	66	3	4	7	10.6
Myanmar	56	3	2	5	8.9
Others	291	28	7	35	12.0
**Health insurance**					
National health insurance	1313	107	48	155	11.8
Social Welfare	18	3	0	3	16.7
Others and unknown	60	7	2	9	15.0
**Regimen**					
INH monotherapy	1309	113	48	161	12.3
RFP monotherapy	78	3	2	5	6.4
Other	4	1	0	1	25.0
**Modes of detection**					
Routine screening	95	10	1	11	11.6
Contact investigation	1101	89	46	135	12.3
At hospital	120	13	3	16	13.3
Other mass screening	29	1	0	1	3.4
Others	46	4	0	4	8.7
**Time between entry to Japan and diagnosis**					
within 2 years	381	22	15	37	9.7
more than 2 years, within 5 years	222	18	15	33	14.9
more than 5 years, within 10 years	74	10	1	11	14.9
10 years or more	199	18	1	19	9.5
Unknown	515	49	18	67	13.0

*INH* isoniazid, *RFP* rifampicin, *LTFU* lost to follow-up

The results of logistic regression analysis, whereby all of the above characteristics were entered as explanatory variables, are summarized in Table 4.

**Table 4:** Risk factors for lost to follow-up and transfer-out combined among foreign-born LTBI patients

	Crude OR	95% CI	Adjusted OR	95% CI	***P***
**Sex**					
Male	Reference				
Female	1.08	0.78–1.49	1.16	0.81–1.66	0.41
**Age groups (age groups)**					
0–14	Reference				
15–19	0.79	0.21–2.88	2.02	0.20–20.39	0.55
20–29	1.06	0.41–2.78	3.06	0.24–38.79	0.39
30–39	1.58	0.60–4.20	5.50	0.42–71.34	0.19
40–49	1.41	0.51–3.88	5.01	0.37–67.1	0.22
50 plus	1.02	0.35–2.94	3.58	0.26–49.37	0.34
**Job status**					
Healthcare workers (HCW)	Reference				
Full-time work, other than HCW	1.17	0.58–2.39	1.29	0.60–2.76	0.51
Self-employed	1.18	0.24–5.84	1.21	0.23–6.39	0.82
Employed on daily/temporal basis	2.67	1.21–5.88	3.11	1.34–7.26	< 0.05
Houseworkers	1.43	0.28–7.20	1.12	0.21–5.89	0.89
Students, high school and above	1.22	0.60–2.50	1.98	0.85–4.64	0.11
Secondary, primary school students, other children and infants	1.25	0.43–3.64	4.97	0.42–58.75	0.20
Unemployed	1.05	0.40–2.79	1.07	0.35–3.26	0.90
Others and unknown	3.14	1.33–7.41	4.09	1.60–10.48	< 0.05
**Country of Birth**					
Others	Reference				
the Philippines	1.17	0.72–1.88	1.05	0.63–1.77	0.84
China	0.99	0.59–1.65	0.92	0.54–1.57	0.75
Vietnam	1.17	0.68–2.00	1.38	0.76–2.49	0.29
Nepal	0.70	0.38–1.29	0.64	0.33–1.24	0.18
Indonesia	0.87	0.37–2.05	1.19	0.48–2.97	0.71
**Regimen**					
RFP monotherapy	Reference				
INH monotherapy	2.05	0.82–5.14	2.14	0.82–5.54	0.12
Other	4.87	0.43–55.7	4.57	0.29–71.53	0.28
**Health insurance**					
National health insurance	Reference				
Social welfare	1.49	0.43–5.22	1.85	0.45–7.61	0.39
Others and unknown	1.32	0.64–2.73	1.13	0.53–2.43	0.76
Modes **of detection**					
Contact investigation	Reference				
Routine screening	0.94	0.49–1.80	1.09	0.54–2.21	0.80
At hospital	1.10	0.63–1.92	1.17	0.63–2.17	0.63
Other mass screening	0.26	0.03–1.89	0.19	0.02–1.47	0.11
Others	0.68	0.24–1.93	0.64	0.22–1.89	0.42
**Time between entry to Japan and diagnosis**					
within 2 years	Reference				
more than 2 years, within 5 years	1.62	0.98–2.68	1.69	1.00–2.87	0.05
more than 5 years, within 10 years	1.62	0.79–3.35	1.50	0.69–3.26	0.31
10 years or more	0.98	0.55–1.76	0.85	0.43–1.68	0.65

*INH* isoniazid, *RFP* rifampicin, *CI* confidence interval, *OR* odds ratio

Variable which remained statistically significant at *P*-value of < 0.05 was being employed on a temporal basis, and job status unknown.

## Discussion

Our results indicated that LTBI notifications among the foreign-born persons have continued to increase, although this is not surprising considering that the number of foreign-born persons from high TB-burden countries entering Japan have continued to increase [9]. It is certainly possible that foreign-born LTBI patients acquired their infection after immigrating to Japan, however, a molecular-epidemiological study which examined the transmission dynamics of TB among foreign- and Japan-born patients in a large urban area in Japan has suggested otherwise, indicating that most of the foreign-born TB cases were attributable to reactivation of LTBI that were acquired in their home country [10]. Among the Japan-born patients, the rise was mainly seen among the older persons, which can be attributable largely due to the abolition of age limit for LTBI treatment in the national guideline in 2010 [11] and an increasing number of elderly persons being tested for and diagnosed with LTBI before being treated for other medical conditions such as rheumatoid arthritis (i.e. hence LTBI being detected “at hospital”). On the other hand, among the foreign-born persons, the LTBI notifications increased in all age groups except those aged between 0 and 14 years old, and among all modes of detection. Among the Japan-born persons, “routine” screening for LTBI has only really been conducted at selected workplaces, such as among high-risk healthcare workers – however, the constant increase in the notification of LTBI among foreign-born persons via “routine screening” may indicate that increasingly, schools and workplaces are making individual decisions to introduce LTBI screening as part of the routine health-check for foreign-born persons. Indeed, there have been sporadic reports of LTBI screening being conducted as part of medical examination upon admissions to universities [12, 13]. In the absence of a clear national guideline on priorities for LTBI among foreign-born persons in Japan, rigorous studies on effectiveness and cost-effectiveness are urgently needed to determine the most appropriate intervention for case finding of LTBI targeting foreign-born persons in Japan.

The majority of the patients had started LTBI treatment with INH monotherapy. This is understandable considering that, although the WHO guideline recommends both INH monotherapy for 6 months for adults and children in countries of both low and high TB incidence, and RFP monotherapy for 3 to 4 months for both adults and children in countries with low TB incidence [7], the Japanese national guideline recommends INH monotherapy above RFP monotherapy [8]. As to why a higher proportion of foreign-born patients were started with RFP monotherapy – we were unable to find an explanation from our results. We may however speculate that physicians in Japan are more inclined to RFP monotherapy because of their knowledge and awareness regarding higher prevalence of INH resistance among foreign-born TB patients [4].

As for the issue of treatment adherence, to our knowledge, this is the first detailed analysis of LTBI treatment outcome among foreign-born persons at a national level in Japan. Outside of Japan, several systematic reviews on adherence for and outcomes of LTBI treatment have been conducted – for example, a review that was conducted for the WHO 2015 LTBI Guideline has concluded that treatment completion rates varied across different risk groups, ranging from 6 to 94%, with lower completion rates for prisoners and immigrant [14]. Another study which looked at studies from US and Canada has reported completion rates to be ranging between 22 and 90% [15], and a more recent review reported rates between 7 to 86%, both for foreign-born person [16]. A meta-analysis that was published in 2016 has estimated the pooled treatment completion rates for migrants to be 14.3% [17]. The studies which were included in these systematic reviews do, however, vary considerably in terms of sample size, study design, types of immigrant and treatment regimen, and hence caution is required when interpreting the results. Our study results indicate the treatment completion rate for foreign-born persons in Japan is relatively high, compared to other studies included in the abovementioned reviews, albeit not reaching the national target of 85%.

Many studies have also examined the predictors for adherence to LTBI treatment, and in general, have tended to conclude that demographic factors such as age, sex, place of birth and race do not seem to influence completion rate [15]. In our study too, neither sex nor age were conclusive risk factors for lost to follow-up among foreign-born LTBI patients. However, as for age, although not statistically significant, there was a clear tendency for the risk to increase with age. This is quite understandable, as younger children are usually overseen by their guardians and are therefore less likely to become lost to follow-up, as well as for the fact that anti-tuberculosis drugs are unusually better tolerated at younger age. Other socio-economic factors, such as unemployment and lack of health insurance, have previously been associated with failure to complete treatment [18, 19] – in our study, none of the foreign-born LTBI patients who had started treatment were reported as “non-insured”. Receiving social welfare assistance and having “others and unknown” for insurance status, which could include short-term visitors with private health insurance, were associated with elevated risk but were not statistically significant. As for job status, being employed on a daily or temporal basis, and having “others and unknown” for job status, were both associated with being lost to follow-up. Considering that LTBI treatment is publicly subsidized with minimum out-of-pocket payment, and is unlikely to place a significant financial burden for the patients, being employed on a daily or temporal basis may represent not necessarily poor economic condition but more an unstable lifestyle and greater mobility which are putting patients at risk of becoming lost to follow-up.

As for the length of treatment regimen, while the WHO 2015 Guideline has concluded that longer treatment duration was detrimental to treatment completion, a systematic review that was published a year later has concluded that study results were inconclusive, with some showing better treatment outcomes for shorter regimens using rifampicin, pyrazinamide, rifabutin and/or INH, than the standard regimen (6 or 9 months of INH) while others showing similar completion rates, and also that the studies themselves were too heterogeneous to conduct pooled analysis [16]. In our study, the proportion of lost to follow-up was bigger among those who have started treatment with INH than those with RFP monotherapy (12.3% vs. 6.4%), however, the difference was shown not to be significant in the logistic regression analysis (*p* = 0.12). There could also be various cofounding factors, and a separate survey may be necessary before conclusions can be drawn. It must also be noted that the information regarding treatment regimen is only of that upon notification – in reality, regimen can and do change during the course of the treatment [9], however, neither the change nor the new regimen after change is captured in the JTBS.

It has been reported that one of the major challenges in ensuring adherence especially for LTBI treatment is to overcome the psychological resistance held by patients to take drugs for a non-contagious and non-symptomatic infection that may never develop into active disease, but which could cause potential adverse effects, and convince them of the potential benefits of prevention [15]. This could be challenging especially when targeting patients who come from culture that is unfamiliar with the concept of screening and prevention [20]. For example, a prospective study that has examined the predictors for non-completion of LTBI treatment has concluded that a perceived risk of progression to active TB is strongly associated with better adherence [21]. However, discussions regarding which factors may influence the construction of such perceived risk and benefit are still inconclusive. One possible factor is the status of being a contact of an active TB patient, and having a close experience with the disease. Indeed, some studies have indicated that contacts tended to have higher completion rates than other population groups [22–24]. In our study, whether or not the patient was a contact of a TB case could be identified from the variable “mode of detection” – those who were detected by contact investigation are obviously case contacts, however, our results indicated that being a contact was not associated with better treatment adherence. This may suggest that being a contact of a TB case alone is a weak motivating factor to adhere to and complete LTBI treatment, at least among foreign-born persons in Japan.

Another potential factor is patient education and counselling – for example, studies from prison have indicated that those who have received education sessions about LTBI prior to being released had higher treatment completion rates post-release, than those who did not [25]. Several studies have shown that well-designed educational intervention using culturally and socially sensitive languages can assist patients make better-informed decisions about the potential risks and benefits of LTBI treatment [26, 27]. We were unable to assess the possible impact of education as such is not collected in the JTBS. However, numerous studies on adherence support to foreign-born active TB patients in Japan have pointed to the challenge of language [28, 29]. Even today, Japan continues to be a largely mono-cultural society and it is quite common that local healthcare workers only speak Japanese. There is an increasing awareness of the need to improve the language capacity of health services, against the rising number of foreign-born patients in Japan – however, the resources are still very much limited. Under such situation, it is not difficult to imagine that foreign-born patients in Japan are not receiving the appropriate and adequate information that they need to make informed decisions about LTBI and treatment. Studies are needed to explore the most effective information, education, and communication for foreign-born patients regarding LTBI.

Finally, we considered the time between entry to Japan and diagnosis of LTBI as a potential risk factor for lost to follow-up. Compared with those who had been diagnosed within 2 years of arriving to Japan, those who had been in Japan for more than 2 years but less than 5 years had a slightly raised risk of becoming lost to follow-up (adjusted odds ratio 1.69, 95% confidence interval 1.00–2.87). The risk probably reflects the risk of transferring-out of Japan, rather than becoming lost to follow-up in Japan, as the proportion of international transfer-out was the highest among those who had been in Japan for more than 2 years but less than 5 years (*n* = 15/222, 6.7%). The reasons are not clear, though this may be related to the duration of permit to stay, which majority of foreign-born students and workers are requested to apply when staying in Japan. The duration of stay depends on the type of permit, however, and it is usually 1 year, 3 years or 5 years. In other words, those who are transferring out of Japan may simply be doing so, because their permit to stay is expiring. This brings up another important issue surrounding the continuum of care for LTBI patients across national borders, but the discussions are beyond the scope of this study.

Our study is not without limitations. Firstly, as our sole source of data was the JTBS, we were unable to examine the effects of variables which were not collected in the JTBS, such as frequency of side-effect [30, 31], and alcohol and/or drug dependence [28, 32], which have previously been identified as being potentially detrimental to treatment adherence. Secondly, our study did not take into account those who have become lost to follow-up at various stages in the cascade of care in diagnosis and treatment of LTBI, including those who, despite being eligible, were not tested for LTBI and those, after being diagnosed as LTBI, did not initiate treatment. For example, the previously mentioned meta-analysis has concluded that screening completion rates was the lowest among the migrants (43%), compared with other populations such as medical personnel (86.1%), marginalized persons (83.3%) and contacts of active case (79.3%) [17]. A separate study is probably required to examine the entire cascade of care for LTBI among foreign-born persons in Japan.

## Conclusions

Our study results have added to the growing evidence that migrant population face greater risk of interrupting LTBI treatment. While the WHO updated guideline for LTBI management states that “concerns about adherence should not be a barrier to use of preventive treatment” [7], the authors nevertheless believe that clinical benefit to individual patient and the success of control programs are subject to completion of LTBI treatment, and hence interventions to improve adherence are a critical component of programmatic management of LTBI.

As previous studies have already shown, efforts to establish a “one-size fits-all” approach to improve adherence to LTBI treatment are probably not likely to succeed. Our results have indicated risk factors for lost to follow-up, some of which were potentially unique to Japan. On the other hand, we have also pointed to the need for a further, in-depth study to explore the socio-economic and cultural situation in which foreign-born persons initiate LTBI treatment in Japan. Whatever the target population, sharing the experience of trying to understand the population and identifying potential barriers, and of innovative approaches to improving adherence can be beneficial to the global effort in tackling LTBI.

## Supplementary Information

**Additional file 1.** Definitions of selected variable from the Japan Tuberculosis Surveillance system.

**Additional file 2.** Annual trend of LTBI notification by country of birth and age groups, 2007–2018. Excel file (.xls) showing the number of LTBI notifications by country of birth and age groups.

**Additional file 3.** Trend of LTBI notification by mode of detection, 2007–2018. Excel file (.xls) showing the number of LTBI notifications by mode of detection, 2007–2018.

## Data Availability

All data generated or analysed during this study are included in this published article [and its supplementary information files].

## Abbreviations

INH: Isoniazid
JTBS: Japan Tuberculosis Surveillance
LTBI: Latent tuberculosis infection
RFP: Rifampicin
TB: Tuberculosis
