# Supplementary material for: Trend and treatment outcomes of latent tuberculosis infection among migrant persons in Japan: retrospective analysis of Japan tuberculosis surveillance data
Source: BMC Infect Dis. 2021 Jan 9;21:42. doi: 10.1186/s12879-020-05712-1 (PMC7796533; doi:10.1186/s12879-020-05712-1)
Supplement: Supplementary file 1 — Additional file 1. Definitions of selected variable from the Japan Tuberculosis Surveillance system. [file 12879_2020_5712_MOESM1_ESM.docx]

**Definitions of the main variables of Japan TB Surveillance System analyzed in this study**

*Country of birth*: Information regarding nationality (either “Japanese” or “non-Japanese”) was added to JTBS in 1998, and country name and the year of entry (either “within five years”, or “more than five years” or “unknown”) in 2007. In 2012, the category of nationality was changed to country of birth (either “Japan-born”, “foreign-born” or “unknown”), and the exact year of entry to Japan. Those whose country of birth was unknown were excluded from the analysis.

*Modes of detection*: JTBS collects data on how the patients initially came to be tested and diagnosed, and are categorized as follows: “private health-check”, “routine school health-check”, “routine community health-check”, “routine workplace health-check”, “routine health-check (others)”, “close contact investigation”, “casual contact investigation”, “other mass screening”, “hospital visit, with TB symptoms”, “hospital visit, with other diseases”, “hospitalized, with other diseases”, “others”, “unknown”, and “health-check during TB treatment follow-up”. For the purpose of this study, these were re-categorized into “routine health-check” (including school, community, workplace and other), “contact investigation” (including close and casual), “at hospital” (including hospital visits and hospitalization”, and “others” (all others).

*Treatment outcomes*: JTBS evaluates treatment outcomes for LTBI in terms of the following categories, namely “treatment successful”, “died”, failure”, “lost to follow-up”, “transferred out”, “still on treatment” and “unknown”. Transfer-out includes both transfers within and outside of Japan.

*Treatment regimens:* In Japan, the Guideline on Treatment of LTBI [7] recommends 6- or 9-months regimen by isoniazid as the first option, followed by 4- or 6-months regimen by rifampicin, which is only recommended when the possibility of the use of isoniazid is ruled out. Information regarding the initial treatment regimen upon notification can be collected from two separate variables, namely “treatment regimen” and “use of rifampicin (RFP)”. “Treatment regimen” is entered as a categorical data, one of which is “isoniazid (INH) monotherapy”. Another category, which is “monotherapy, other than INH”, does not in itself provide information regarding which drug was prescribed. However, “use of RFP”, which is entered as “yes” or “no”, can be used to indicate whether RFP was used for “monotherapy, other than INH”. It must be noted that in real settings, the regimen can and does change during the course of treatment, however, neither the change nor the new regimen is captured in the JTBS. Other options for treatment regimen include the standard treatment regimens for active TB, as well as “no treatment” and “unknown”. For the purpose of this study, all other categories, excluding “no treatment” were recategorized as “others”.
